# Gut mycobiome and its interaction with diet, gut bacteria and alzheimer's disease markers in subjects with mild cognitive impairment: A pilot study

**DOI:** 10.1016/j.ebiom.2020.102950

**Published:** 2020-08-30

**Authors:** Ravinder Nagpal, Bryan J. Neth, Shaohua Wang, Sidharth P. Mishra, Suzanne Craft, Hariom Yadav

**Affiliations:** a Department of Internal Medicine-Molecular Medicine, Wake Forest School of Medicine, Winston-Salem, NC, United States; b Department of Microbiology and Immunology, Wake Forest School of Medicine, Winston-Salem, NC, United States; c Department of Internal Medicine- Gerontology and Geriatric Medicine, Wake Forest School of Medicine, Winston-Salem, NC, United States; d Department of Neurology, Mayo Clinic, Rochester, MN, United States

**Keywords:** Alzheimer's, Dementia, Fungi, Mycobiota, Microbiome, Nutrition, Ketogenic diet, Mediterranean diet, Short-chain fatty acids, amyloid peptides, tau, AD:, Alzheimer disease, AHAD:, American Heart Association Diet, Aß:, amyloid beta, ApoE ε−4:, apolipoprotein-E ε−4 allele, CN:, cognitively normal, CSF:, cerebrospinal fluid, ITS:, internal transcribed spacer, KD:, ketogenic diet, LDA:, linear discrimination analysis, LEfSe:, linear discrimination analysis effect size, LP:, lumbar puncture, LPS:, lipopolysaccharide, MCI:, mildly cognitive impairment, MMKD:, modified Mediterranean-ketogenic diet, OTUs:, operational taxonomic units, PCoA:, principal coordinate analysis, SCFAs:, short-chain fatty acids

## Abstract

**Background:**

Recently, we reported that patients with mild cognitive impairment (MCI) harbor specific signature of bacteria in their gut and that a modified Mediterranean ketogenic diet (MMKD) improves Alzheimer's disease (AD) markers in cerebrospinal fluid (CSF) and the signatures of gut bacteria. However, other microbial population such as gut fungi (mycobiome) in relation to MCI/AD pathology, gut bacteria and diet remain unknown.

**Methods:**

We measure gut mycobiome by sequencing of the fungal rRNA ITS1 gene in 17 older adults (11 MCI; 6 cognitively normal [CN]) in a single-center, randomized, double-blind, crossover pilot study, before and after 6 weeks intervention of MMKD and American Heart Association Diet (AHAD), and determine its correlation with AD markers in CSF and gut bacteria.

**Findings:**

Compared to CN counterparts, patients with MCI have higher proportion of families *Sclerotiniaceae, Phaffomyceteceae, Trichocomaceae, Cystofilobasidiaceae, Togniniaceae* and genera *Botrytis, Kazachstania, Phaeoacremonium* and *Cladosporium* and lower abundance of *Meyerozyma*. Specific fungal taxa exhibit distinct correlation arrays with AD markers and gut bacteria in subjects with versus without MCI. MMKD induces broader effect on fungal diversity in subjects with MCI and increases *Agaricus* and *Mrakia* while decreasing *Saccharomyces* and *Claviceps* with differential response in subjects with or without MCI.

**Interpretation:**

The study reveals MCI-specific mycobiome signatures and demonstrates that distinct diets modulate the mycobiome in association with AD markers and fungal-bacterial co-regulation networks in patients with MCI. The findings corroborate the notion of considering gut mycobiome as a unique factor that can affect cognitive health/AD by interacting with gut bacteria and diet and facilitate better understanding of the AD and related microbiome, using unique diet or microbiome modulators.

## Graphical abstract

**Figure:**
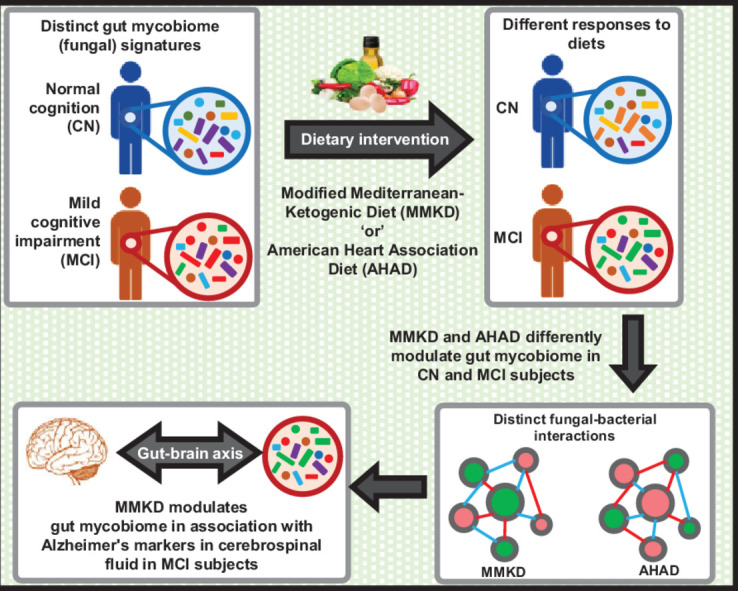

**Research in context**

**Evidence before this study**

Despite extensive research in understanding the genetic and molecular risk factors of Alzheimer's disease (AD) pathogenesis, there is currently no established therapy to prevent or ameliorate AD pathology. Specific dietary patterns can be an efficient mean to prevent or delay the progression of the disease. Compared to cognitively normal counterparts, patients with mild cognitive impairment harbor distinct gut bacterial signatures that correlate with AD's pathological markers in the cerebrospinal fluid (CSF) and could play a potential role in AD pathogenesis. A modified Mediterranean-style ketogenic diet (MMKD) has been found to improve the profiles of CSF AD biomarkers and modulate the gut bacterial microbiome in patients with MCI.

**Added value of this study**

This is the first study to examine the gut fungal microbiome (mycobiome) in patients with MCI while also evaluating the effect of MMKD on gut mycobiome in association with CSF AD biomarkers and gut fungal-bacterial co-occurrence network. The study identifies MCI-specific gut mycobiome signatures and demonstrates how these signatures correlate with gut bacteria as well as with CSF AD biomarkers including the deposition of β-amyloid (Aβ)−40, Aβ−42 and total and phosphorylated tau. The study also demonstrates how MMKD distinctly modulates the gut mycobiome and fungal-bacterial co-regulation networks in associations with the CSF AD biomarkers in subjects with or without MCI.

**Implications of all the available evidence**

Our growing understanding of the role of gut microbiome in AD pathobiology − irrespective of whether the role is causative or consequence − could open novel hypotheses in the field and inspire further investigation of potential gut microbial signatures and associated dysbiosis and dysfunctions of gut-brain axis that might contribute to dementia, cognitive decline and AD progression in high-risk subjects. Gut microbiome may be one of the factors to consider in the prevention and/or amelioration of AD and related dementia and cognitive impairment particularly in the elderly.

Alt-text: Unlabelled box

## 1 INTRODUCTION

Alzheimer's disease (AD) is a progressive and fatal neurodegenerative disorder that affects over 40 million people worldwide and is the leading cause of dementia and cognitive decline particularly in older adults [1]. Despite extensive research on genetic and molecular risk factors of AD pathogenesis, no therapy is currently available to prevent or slow AD progression, which underlines the complexity and our inadequate knowledge of disease mechanisms while also stressing the need for exploring novel therapeutic targets and unconventional interventions to control this devastating condition. AD pathology is characterized by extracellular accumulations of amyloid-β-peptides (Aβ) in the senile plaques, and by intracellular depositions of hyper-phosphorylated tau proteins that forms neurofibrillary tangles [2]. Genetically, subjects carrying Apolipoprotein E (APOE) allele ε−4 are at higher risk for AD as compared to subjects with ε3 and ε2 alleles [3]. However, emerging evidence shows that the AD pathogenesis is much more complex than previously believed and that the brain amyloid deposition might antedate clinical indications by 10–20 years [4]. Studies have shown that AD involves chronic inflammation in the central nervous system (CNS) as well as in the periphery and that the amyloid plaques are consequence of years of chronic inflammation [5], [6], [7]. To the contrary, mild cognitive impairment (MCI) is characterized by a state of cognitive deterioration (with relatively preserved activities of daily living) that precedes the clinical symptoms of dementia and AD [8, 9]. Individuals with MCI are at an increased risk for developing dementia and AD [10, 11] and hence interventions during MCI stages are important for ameliorating AD progression.

Recent multiple evidences suggest that gut microbiome play an important role in the pathogenesis of neurological disorders [12], [13], [14], [15], [16], [17], [18]. Studies have shown the implication of gut-brain axis − an integrated network wherein microbiome and the central nervous system crosstalk via endocrine, immune, and neural signaling pathways − in various aspects of host health and diseases [19, 20]. Recent studies by others and us have reported an altered gut microbiome in patients with MCI, dementia and AD [21], [22], [23], [24], [25], [26], [27], [28] as well as in multiple AD animal models [29], [30], [31], [32]. These evidences suggest that the gut microbiome is linked with host neurological function via gut– microbiome–brain axis [33]. However, whether and how the gut microbiome impacts host cognitive function remain unclear. Bacterial lipopolysaccharide (LPS), a pro-inflammatory component of the cell wall of gram-negative bacteria, has been detected in the brain of AD patients and is speculated to be able to induce systemic inflammation and amyloid accumulation thereby leading to amyloid deposition [34, 35]. In addition, aberrations in the functional pathways comprising circulation, neuroendocrine, neural, and neuro-immune signaling pathways through which the gut microbes and the brain cross-talk with each other are hypothesized as a link between the gut microbiome and host cognitive functions [36], however, the mechanisms are not defined. Hence, investigation of the gut microbiome in patients with MCI may prove to be important in identifying previously unknown risk factors during early stages of AD, and thus can be targeted to prevent the AD progression.

Multiple failures of clinical trials for AD treatments focused on single targets indicate that along with complex nature of the AD, the strategies targeting pleotropic effects may be more effective in preventing and ameliorating the AD pathology. For example, the lifestyle modifications including diet and exercise are only effective strategies currently known to reduce the risk of AD, and both of these are superlative modulators of gut microbiome [22] and hence may prove to be key public health preventative approaches. Diet is one of the most prominent modulator of the gut microbiome and is known to prevent aging-related microbiome alterations [37]. Studies using animal models of aging have reported that a calorie-restricted low-carb diet without malnutrition promotes healthy aging [38], stimulates neuroprotective signatures [39], prevents aging-related transcriptional changes in the hippocampus [39], prevents accumulation of amyloid-beta (Aβ) plaque by modulating the expression of Aβ precursor protein [40], and also modulates the microbiome by fostering specific gut microbes associated with healthy aging [41]. Cerebral amyloidosis and severe tauopathy in brain are crucial pathological characteristics of AD. Despite emerging evidence linking gut microbiome with AD, the association between specific dietary patterns, gut microbes, and AD pathophysiology remains unclear. Recently, in a study on older adults clinically diagnosed with mild cognitive impairment (MCI) and their cognitively normal (CN) counterparts, we reported that a modified Mediterranean-style ketogenic diet (MMKD) is well tolerated with good compliance in these older adults at risk for AD [42]. In addition, we also reported that, in comparison to a six-week intervention of a control low-fat American Heart Association Diet (AHAD), an equivalent intervention of MMKD (a) improves the profiles of Alzheimer's biomarkers in the cerebrospinal fluid (CSF), (b) increases the cerebral perfusion and cerebral ketone body uptake and metabolism, and (c) improves metabolic health in patients with MCI. Concurrently, we also reported specific gut bacterial microbiome signatures in patients with MCI [24], wherein the MMKD was found to modulate the gut bacterial population in association with improved profiles of CSF markers of AD in patients with MCI. However, recent evidence indicates that the diet-microbe association in the otherwise complex human intestinal microbial ecosystem may not exclusively be limited to a particular microbial kingdom (bacteriome) but instead might comprise inter-relationships with other microbes such as fungi (mycobiome) that play a shared role in various milieus of host health and disease. However, whether and how the gut mycobiome differ between patients with MCI versus healthy counterparts and whether and how specific dietary interventions affect these mycobiome signatures remain unknown. Given the high prevalence of fungi in our environment, it is unsurprising that fungi also constitute part of the human microbiome. Although the mycobiome represent a relatively small fraction of the human microbiome, recent studies have begun to examine their role in human health and disease. Studies have implicated the role of gut fungi in several human diseases including inflammatory bowel disease [43, 44], enterocolitis [45, 46], colorectal cancers [47], graft versus host disease [48], alcoholic liver disease [49], asthma [50] and hepatitis B virus infections [51]. In addition, several studies have reported a dysbiotic gut fungal microbiota in patients with Autism spectrum disorder [52], [53], [54]. These studies underline the need to examine the gut mycobiome more broadly, which would help in identifying potential disease-contributing fungal clades while uncovering fungal-bacterial relationships important for host health. Here, in this addendum, we for the first time demonstrate distinct gut mycobiome signatures in patients with MCI, along with potential interactions of the gut mycobiome signatures with host diet, gut bacteria and CSF AD biomarkers.

## 2 METHODS

### 2.1 Study details

#### 2.1.1 Study design

The trial was registered prior to the recruitment commencement (Clinical Trials # NCT02984540). The study was approved by the Institutional Review Boards of the Wake Forest School of Medicine and was conducted in the Wake Forest Clinical Research Units. All protocols related to the cohorts involved in the study were reviewed and approved by the Institutional Review Board of the Wake Forest School of Medicine. Details about the study design, participants, recruitment, inclusion/exclusion criteria, and dietary intervention have been provided in our previous reports [24, 42]. Briefly, the study was a randomized, double-blind, crossover, single-center pilot trial that included 17 subjects (mean age: 64.6 ± 6.4 yr) of which 11 participants were diagnosed with mild cognitive impairment (MCI) on the basis of ADNI-2 criteria for early MCI (http://www.adni-info.org) and at risk for AD due to baseline MCI and cogni/subjective memory complaints. The remaining six participants were cognitively normal (CN). The primary goal of the original study was to examine the effects of MMKD and AHAD on CSF AD biomarkers, neuroimaging measures, peripheral metabolic measures, and cognition, as already published elsewhere [42].

#### 2.1.2 Study participants

Written informed consent was obtained from all study participants and/or their study partners or legal representatives. Participants were medically monitored by the clinicians, and safety supervision was monitored by the Wake Forest Institutional Data and Safety Monitoring Committee as described previously [42]. The study was originally projected and designed for the recruitment of 30 participants for the dietary intervention study, with at least 20 subjects completing both diets at study completion, thereby generating a greater sample size than that used in other studies reporting the beneficial effect of a 6-week ketogenic diet intervention on verbal memory in MCI subjects [55] and therefore also increasing the power to detect the diet's effect in the study groups. In addition, the crossover design of the study increased the power and reduced the outcome variability in that each subject served as their own control for the individual effects of diets. The present study finally included only those 17 subjects that were able to provide stool specimen at each time-point of the two dietary arms. Exclusion criteria included previous diagnosis of neurological/ neurodegenerative disorder (other than MCI), stroke, major psychiatric disorder, use of anti-diabetic and lipid-lowering medication, or medications with known central nervous system effects i.e., anti-seizures, anti-psychotics, and opioids. Subjects with well-controlled depression were permitted.

#### 2.1.3 Procedure

All subjects underwent a lumbar puncture (LP) at the baseline and end of diet 1 and again at the end of diet 2. Fresh fecal specimens were collected at baseline and end of each diet arm. All experimental procedures and samplings were executed in accordance with ethical and biosafety protocols approved by the Institutional guidelines. All study personnel involved in collecting and analyzing the data remained blinded to MCI status or diet intervention allocation throughout the study. Diagnoses, eligibility and inclusion/exclusion criteria were evaluated by expert physicians and neuropsychologists based on the data of cognitive testing, medical history, physical examination and clinical laboratory, as per the criteria described previously [42].

### 2.2 Dietary intervention

Participants were randomly assigned to either a modified Mediterranean-style ketogenic diet (MMKD) or an American Heart Association Diet (AHAD) intervention for six-weeks followed by a six-week washout period in which participants were instructed to resume their pre-study diet, following which the second diet was consumed for six weeks. Simple randomization was implemented by a registered dietitian at the beginning of the study. Daily meal plans were formulated by the study dietitian on the basis of food preferences and caloric needs. Diets were designed with the following structure: (1) MMKD: The low-carbohydrate diet consisted of a diet plan of not more than 20 gs of carbohydrates per day to be consumed over the six-week period. Foods with high levels of healthy fats (and low in saturated fats) were generously incorporated in the meal plan. Various lean meats, fish, and nutrient-rich foods meeting the criterion of under 20 gs total carbohydrates per day were included in the diet plans. Carbohydrates were estimated to make up for not more than 10% of total caloric intake. Subjects on this diet were provided with 2 liters of extra virgin olive oil at study visits to incorporate into their individualized meal plans. (2) AHAD: The low-fat diet consisted of a low-fat, high-carbohydrate diet plan to be consumed over the 6 weeks. Subjects were encouraged to restrict the amount of fat intake to not more than 40 gs per day, while eating ample fruits, vegetables, and adequate carbohydrate-containing fiber. Various lean meats and other protein sources were included in the plan. Carbohydrates were estimated to make up for 50–60% of total caloric intake. Overall, the intended macronutrient composition (% of total calories) was <10% carbohydrate, 60–65% fat and 30–35% protein for the MKMD, and 55–65% carbohydrate, 15–20% fat and 20–30% protein for the AHAD. While on the MKMD, subjects received extra virgin olive oil and were encouraged to eat fish, lean meats, and nutrient rich foods. While on the AHAD, subjects were directed to restrict their fat intake to <40 gs/day while eating fruits, vegetables and fiber-containing carbohydrates.

### 2.3 Fecal bacterial and fungal microbiome analysis

#### 2.3.1 Microbiome measurement

The bacterial and fungal microbiome were analyzed according to the Earth Microbiome Project (EMP) benchmarked protocol [56] (http://www.earthmicrobiome.org), by employing a barcoded high-throughput sequencing approach as described by Caporaso et al. [57]. Briefly, the fecal samples were stored at −80 °C as soon as possible after collection until microbial DNA extraction. To avoid the influence of DNA extraction, PCR conditions and primers on community composition recovered by amplicon sequencing, all samples were processed simultaneously and identically in order to minimize biasing the fungal community composition. The details of fecal bacterial (16S rRNA gene) sequencing, analysis and diet-effects have been provided in our previous report [24]. The fungal amplicon libraries were prepared using the primer pair targeting the internal transcribed spacer (ITS), a region of the nuclear ribosomal RNA cistron known to allow effective identification across a broad range of fungal taxa [58]. The DNA was extracted using the MoBio PowerFecal kit (Qiagen, CA, USA) as per the manufacturer's instructions, and the first internal transcribed spacer region (ITS1) of the fungal rRNA gene was amplified using the ITS1f (5′-CTTGGTCATTTAGAGGAAGTAA) and ITS2 (5′-GCTGCGTTCTTCATCGATGC) primer pair. Both the forward and reverse primers also had appropriate Illumina adapters, primer pad, and 2-bp linker sequences with the reverse primer containing a 12-bp error-correcting barcode unique to each sample to achieve multiplex capability. All primers were synthesized at the Integrated DNA Technologies (IDT Inc., San Jose, CA, USA). The PCR amplification reaction was performed as described previously [24, 59]. The resulting amplicons were purified using Agencourt® AMPure® XP magnetic purification beads (Beckman Coulter, Brea, CA, USA); quantified using the Qubit-3 fluorimeter (InVitrogen, Carlsbad, CA, USA) and dsDNA HS assay kit (Life Technologies, Carlsbad, CA, USA); and the amplicon library was generated according to methods of Caporaso et al. [57]. The purified PCR product was pooled in equal molar concentrations and sequenced on one 2 × 300-bp Illumina MiSeq run (Illumina Inc., San Diego, CA, USA) for paired-end sequencing. Negative controls were mock samples that contained all reagents, including barcoded primers but without any extracted DNA that went through PCR and sequencing processes.

#### 2.3.2 Microbiome analysis

The sequencing quality control was executed with on-board Miseq Control Software and Miseq Reporter (Illumina Inc., San Diego, CA, USA). The resultant paired-end reads were demultiplexed and were assigned to individual samples based on their unique barcode. The taxonomic classification of fungal rRNA targeted amplicon reads was performed by using the Illumina Fungal ITS Metagenomics workflow (Illumina Inc.) using default parameters. Briefly, the naive-Bayes high-performance implementation of the RDP (Ribosomal Database Project)-classifier algorithm was performed as described by Wang et al. [60]. The taxonomic identification was performed on the basis of clustering at 97% similarity level against the UNITE Fungal database (alpha-version 7.2, 2017 release; https://plutof.ut.ee/#/doi/10.15156/BIO/587481) for the fungal ITS1 region [61]. A total of 2399,527 sequences were obtained of which 1842,661 sequences (mean 21,678 sequences/sample) were retained after quality-filtering using default parameters. To avoid the bias of sequencing errors or low-level contaminations, the OTUs with very small read count (less than 4) in very few samples (less than 10% prevalence) were filtered out from the subsequent analyses. The data of taxon abundance were subjected to the total sum scaling and the taxa with less than 1% mean relative abundance were further excluded from the subsequent downstream analyses. Fungal community composition of each sample was measured at taxonomic levels of phyla, classes, orders, families and genera.

### 2.4 Lumbar puncture, CSF biomarkers assays, and ApoE ε−4 genotyping

The procedures and analysis of lumbar puncture, CSF biomarkers assays, and ApoE ε−4 genotyping have been detailed elsewhere [42, 62]. Briefly, the CSF (25 ml) was collected using a 20 gage Sprotte spinal needle and polypropylene tubes (as per the standardized best practice guidelines of the National Alzheimer's Coordinating Center) in the morning times after an over-night fast. CSF was aliquoted (200 µL) into pre-chilled polypropylene tubes, frozen on dry ice, and stored at −80 °C. AD biomarkers (Aβ−42, Aβ−40, tau and tau-p181) were measured by using the AlzBio3 multiplex assay (FujiRebio, Europe). Subjects were stratified on the basis of the carriage of the ApoE ε−4 allele, a well-established risk factor for AD that predicts insulin response, per our previously described method [63].

### 2.5 Fecal lactate and SCFAs analyses

Fecal concentrations of lactate and short-chain fatty acids (SCFAs) including acetate, propionate and butyrate were measured using a high-performance liquid chromatography (HPLC; Waters-2695 Alliance HPLC, MA, USA) system as described in our previous report [24].

### 2.6 Data analysis

The beta-diversity of the mycobiome was assessed using the Bray-Curtis dissimilarity index and was visualized with the principal coordinate analysis (PCoA) in R statistical software package (version 3.6.1; www.r-project.org). Alpha diversity measures included species richness (number of OTUs) and evenness (Shannon index). Random forest supervised learning model was applied on normalized data within R (model_randomForest; 70% training and 30% testing modules; trees = 500) to identify the major fungal taxa whose abundance is affected by the MMKD and AHAD intervention. The subsequent statistical significance of taxon abundance (and alpha-diversity) was assessed with two-tailed unpaired Student's *t*-test when comparing two sample groups or with analysis of variance (ANOVA) in case of more than two groups. Fungal taxa uniquely modulated by MCI status and dietary interventions and driving differences between different groups were determined by using the biomarker discovery algorithm LEfSE (Linear discriminatory analysis [LDA] Effect Size) with LDA score >2.0 and p-value <0.05 [64]. Spearman correlations of fungal taxa with CSF biomarkers and fecal bacterial taxa were calculated in GraphPad Prism software (version 6.0). Co-occurrence networks between fungal and bacterial taxa were calculated by using the open-source software Gephi (version 0.9.2; www.gephi.org). Modularity-based co-occurrence networks between fungal and bacterial taxa were analyzed at a Spearman correlation cutoff of 0.5 and p-value <0.05. Volcano plots depicting log_2_-fold changes in the fungal taxa during the dietary interventions were created within R. Hierarchical clustering heat-maps were constructed within R using the ‘heatmap.2′ package. Unless otherwise stated, all the values presented herein are mean ± SEM. *P*<0.05 was considered statistically significant unless specified.

## 3 RESULTS

### 3.1 Specific gut mycobiome signatures associated with host MCI status

**Fig. 1:**
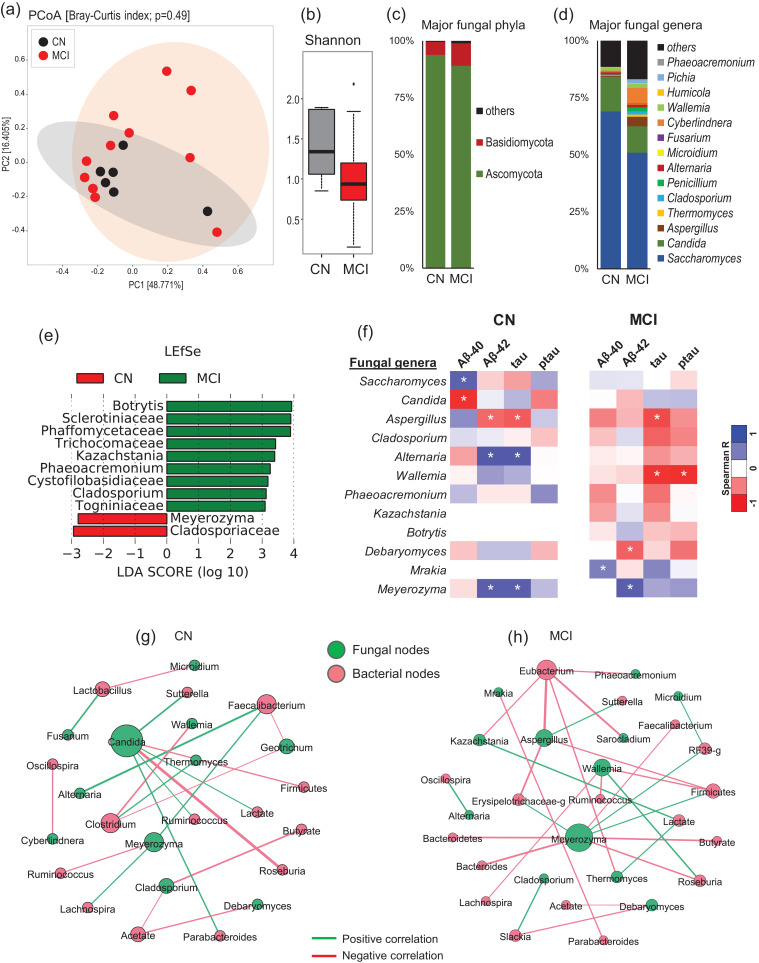
Differences in the gut mycobiome between subjects clinically diagnosed with mild cognitive impairment (MCI) versus cognitively normal (CN) counterparts. (a) Mycobiome β-diversity, (b) α-diversity (Shannon index), (c-d) mycobiome composition at the level of major phyla (c) and genera (d) in CN subjects (*n* = 6) and patients with MCI (*n* = 11). (e): Linear discriminant analysis (LDA) effect size (LEfSe) plot representing the significantly unique fungal taxa identified in CN subjects versus patients with MCI. (f) Correlation (Spearman; *p < 0.05) of gut fungal genera with cerebrospinal fluid markers of Alzheimer's disease in CN subjects versus patients with MCI. (g-h) Ecological co-occurrence network among differentially abundant fungal and bacterial genera in patients with MCI compared with CN subjects. Networks represent statistically significant correlations (Spearman rho >0.5; p-value <0.05).

The analysis of beta-diversity and alpha-diversity of the gut mycobiome at the baseline do not differ significantly between patients with MCI versus CN counterparts (Fig. 1a, b). However, the subjects with MCI show slightly lower fungal species diversity compared to the CN counterparts (Fig. 1b). Compared to CN subjects, the patients with MCI harbor insignificantly lower proportion of phylum Ascomycota and higher proportion of phylum Basidiomycota (Fig. 1c). Accordingly, further analysis at the level of major fungal genera show clearly distinct mycobiome composition in patients with MCI versus CN subjects (Fig. 1d). Linear discriminatory analysis (LDA) effect size (LEfSe) analysis reveals several fungal taxa that are significantly higher or lower in MCI subjects compared to CN subjects (Fig. 1e). Compared to CN subjects, patients with MCI have significantly higher proportion of fungal families Sclerotiniaceae, Phaffomyceteceae, Trichocomaceae, Cystofilobasidiaceae, Togniniaceae and genera *Botrytis, Kazachstania, Phaeoacremonium* and *Cladosporium* while having lower proportion of family Cladosporiaceae and genus *Meyerozyma* (Fig. 1e). Further correlation analysis demonstrates several fungal genera that correlate differently with AD markers in the CSF of MCI versus CN subjects (Fig. 1f). Genera *Saccharomyces* and *Candida* correlate positively and negatively, respectively, with Aβ−40 in CN subjects but not in patients with MCI. *Aspergillus* and *Meyerozyma* correlate negatively and positively, respectively, with Aβ−40 and total tau in both CN subjects and subjects with MCI. *Alternaria* correlates significantly positively with Aβ−40 and tau in CN subjects but correlates slightly negatively with these markers in subjects with MCI. Similarly, *Wallemia* correlates significantly negatively with total tau and tau-p181 in patients with MCI but not in CN subjects. *Debaryomyces* correlates negatively with Aβ−40 while *Mrakia* correlates positively with Aβ−40 in patients with MCI but not in CN subjects. Further fungal-bacterial correlation analysis reveals clearly distinct co-occurrence networks in patients with MCI versus CN subjects (Fig. 1g, h). Interestingly, different fungal ‘keystone’ taxa, which could be defined as organisms that might play a central role in the overall intestinal microbial community stability and function, are seen in patients with MCI versus CN subjects. In patients with MCI (Fig. 1h), *Meyerozyma, Wallemia* and *Aspergillus* are among the main central taxa that correlate with several bacterial taxa including Firmicutes*, Bacteroides, Roseburia,* Erysipelotrichae and *Eubacterium*. Among bacteria, *Eubacterium,* Firmicutes*, Roseburia* and *Slackia* are among the major bacterial taxa associated with several fungal taxa. On the other hand, in CN subjects (Fig. 1g), *Candida* is the central fungal taxon followed by *Meyerozyma, Geotrichum* and *Cladosporium* that are linked to several bacterial taxa including *Faecalibacterium, Sutterella,* Firmicutes*, Roseburia, Parabacteroides, Clostridium, Ruminococcus* and *Lachnospira. Faecalibacterium* and *Clostridium* are among the major bacterial taxa that are centrally correlated to several fungal nodes. Overall, the co-occurrence network in CN subjects show fewer connections (Fig. 1g) as compared with the networks in patients with MCI, which clearly demonstrate a greater density of networks between nodes (Fig. 1h).

### 3.2 MMKD and AHAD differently modulate gut mycobiome composition in patients with or without MCI

**Fig. 2:**
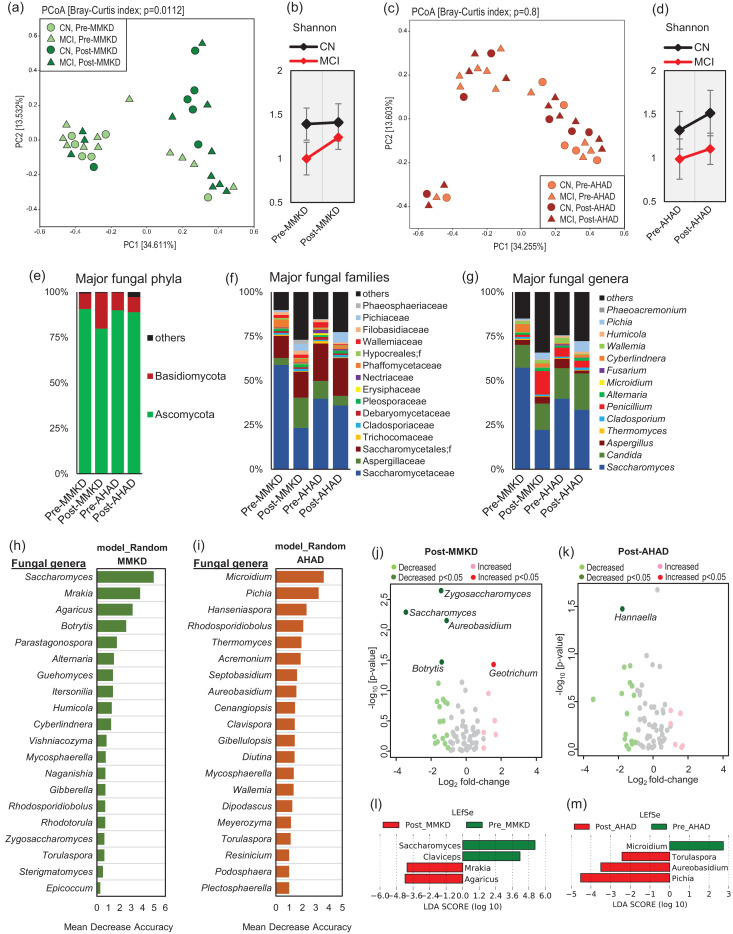
A modified Mediterranean-style ketogenic diet (MMKD) and the American Heart Association Diet (AHAD) differently modulate gut mycobiome in subjects clinically diagnosed with mild cognitive impairment (MCI) versus cognitively normal (CN) counterparts. (a,c) Mycobiome β-diversity before and after MMKD (a) and AHAD (c) in CN subjects and patients with MCI. (b,d) Mycobiome α-diversity before and after MMKD (b) and AHAD (d) in CN subjects and patients with MCI. Mycobiome composition at the level of major phyla (e), families (f) and genera (g) at the baseline and endpoint of 6-weeks MMKD and AHAD intervention. (h-i) The random forest graph showing the 20 most predictive fungal genera influenced by MMKD or AHAD intervention. (j-k) Volcano plots showing the fungal genera significantly increased or decreased in terms of Log_2_-fold change during MMKD or AHAD intervention. (l-m) LEfSe plots representing the fungal genera significantly unique at baseline versus endpoint of MMKD or AHAD intervention.

The six-week intervention of MMKD and AHAD does not produce remarkable changes in terms of the fungal beta-diversity signatures; however, the overall effect of MMKD is more prominent (*p* = 0.0112) on the mycobiome as compared to that of AHAD (*p* = 0.8), indicating that MMKD intervention modulated the mycobiome more profoundly and in more subjects as compared to AHAD (Fig. 2a,c). In terms of alpha-diversity of the fungal community, interestingly, the MMKD intervention does not bring about any change in the Shannon index in CN subjects but induces insignificant but considerable increase in patients with MCI (Fig. 2b). On the other hand, AHAD slightly increases the Shannon diversity in both CN and MCI subjects (Fig. 2d). In line with these data, the mycobiome composition at phylum, family and genus level is modulated more prominently during the MMKD intervention as compared to during AHAD intervention (Fig. 2e-g). Particularly, the composition at genus level is clearly distinct in post-MMKD versus pre-MMKD time-points, whereas the composition at pre-AHAD and post-AHAD time-points is relatively similar (Fig. 1g). For unbiased identification of the discriminatory fungal taxa associated with specific dietary intervention, we apply supervised machine learning-based random forest model trained with fungal genera that also reveals a clearly distinct group of fungal taxa affected by MMKD versus AHAD intervention (Fig. 2h-i). Further analysis of log_2_-fold changes in the proportion of these taxa during dietary intervention also reveals more prominent effect of MMKD versus AHAD on fungal genera (Fig. 2j-k). The abundance of *Saccharomyces, Zygosaccharomyces, Aureobasidium* and *Botrytis* is significantly decreased while that of *Geotrichum* is increased during MMKD (Fig. 2j); whereas, AHAD shows only one significant change i.e., decreased proportion of *Hannaella* (Fig. 2k). The decreased proportion of *Botrytis* during MMKD is interesting because its abundance was significantly higher in subjects with MCI at the baseline (Fig. 1e), again hinting toward a positive effect of MMKD on fungal flora particularly in patients with MCI. The LEfSe analysis that integrates analysis of variance (Kruskal-Wallis test) followed by Wilcoxon rank-sum test and linear discriminatory analysis reveals decreased proportion of *Saccharomyces* and *Claviceps* and increased abundance of *Mrakia* and *Agaricus* during MMKD (Fig. 2l) and decreased proportion of *Microidium* and increased abundance of *Pichia, Aureobasidium* and *Torulospora* during AHAD (Fig. 2m).

**Fig. 3:**
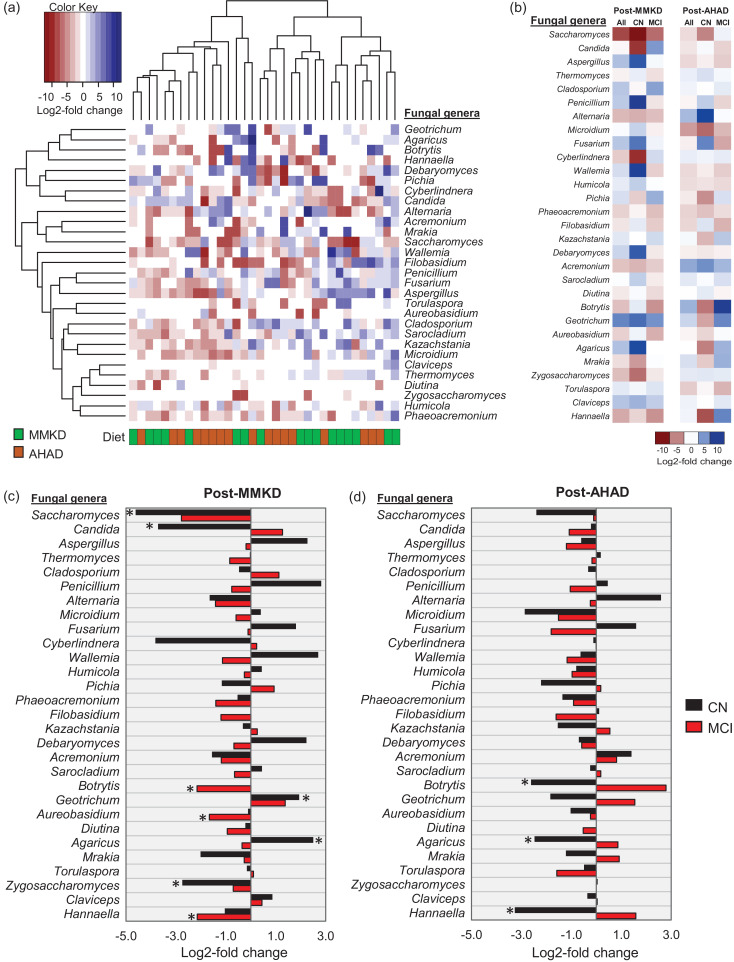
A modified Mediterranean-style ketogenic diet (MMKD) and the American Heart Association Diet (AHAD) induce specific changes in the gut mycobiome of subjects clinically diagnosed with mild cognitive impairment (MCI) versus cognitively normal (CN) counterparts. (a) Hierarchical clustering heat-map of major fungal genera showing Log_2_-fold increase or decrease in relative abundance during MMKD or AHAD intervention. (b) Heat-map summarizing the differential patterns of diet-induced alterations (mean Log_2_-fold change in relative abundance) in major gut fungal genera during MMKD or AHAD intervention in CN subjects and patients with MCI. (c-d) Mean Log_2_-fold change in the relative abundance of major fungal genera in CN subjects (n = 6) versus patients with MCI (n = 11) during MMKD (c) and AHAD (d) intervention (*p < .05).

The hierarchical clustering of these changes in taxon abundance does not demonstrate distinct clusters of the two diets (Fig. 3a); however, we find several taxa that are changed differently in patients with MCI versus CN subjects (Fig. 3b). As further summarized in Fig. 3c-d, *Saccharomyces, Zygosaccharomyces* and *Candida* are significantly decreased on MMKD but not on AHAD and only in CN subjects but not in patients with MCI. Interestingly, *Botrytis*, which was higher in MCI patients at the baseline, is decreased in MCI patients but not in CN subjects on MMKD whereas, in subjects on AHAD, its proportion is decreased in CN subjects and increased in MCI patients. *Geotrichum* and *Agaricus* are increased in CN subjects but not in MCI patients on MMKD but are decreased in CN subjects but not in MCI patients on AHAD. During MMKD, the genus *Hannaella* is decreased only in MCI patients whereas, during AHAD, it is decreased only in CN subjects. These distinct fungal patterns indicate that the two diets differently influence the gut mycobiome and that these influences may further differ according to the host MCI status.

### 3.4 Diet-induced changes in the gut mycobiome correlate with AD markers in the CSF

**Fig. 4:**
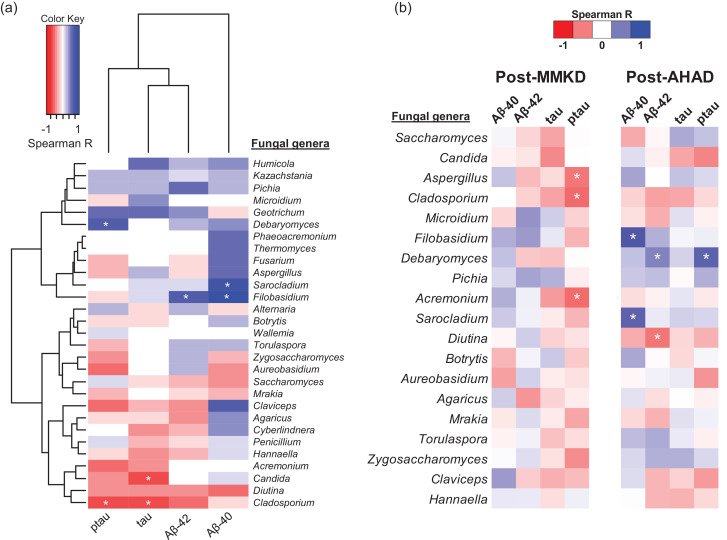
Diet-induced changes in gut mycobiome are associated with changes in cerebral spinal fluid (CSF) biomarkers of Alzheimer's disease (AD) in subjects clinically diagnosed with mild cognitive impairment (MCI) versus cognitively normal (CN) counterparts. Heat-map depicting the (a) overall correlation patterns (Spearman rho; *p < .05) of changes (Log_2_-fold change) in major gut fungal genera with changes in cerebrospinal fluid markers of Alzheimer's disease during (b) MMKD versus AHAD intervention.

The changes occurring in the CSF markers of AD in subjects on MMKD or AHAD have been published in our recent report [42]. Herein, we integrate the data of gut mycobiome with that of CSF markers to identify fungal taxa that correlated with the CSF markers. As shown in Fig. 4a, we find several fungal genera including *Debaryomyces, Sarocladium, Filobasidium, Candida* and *Cladosporium* that correlate positively or negatively with different CSF markers of AD. However, further parsing of these data according to the diet and MCI status reveal that specific correlation patterns that vary between CN versus MCI subjects and between MMKD versus AHAD intervention (Fig. 4b). Interestingly, *Aspergillus* and *Cladosporium*, which were higher in patients with MCI at the baseline, correlate negatively with p-tau during MMKD intervention. The genus *Acremonium* also showed similar spectrum of correlation during MMKD. Whereas, in subjects on AHAD, genera *Filobasidium* and *Sarcoladium* correlate positively with Aβ−40 while *Debaryomyces* and *Dilutina* correlate positively and negatively, respectively, with Aβ−40 (Fig. 4b). In addition, *Debramyces* also correlate positively with p-tau during the AHAD intervention.

### 3.5 MMKD and AHAD induce distinct gut fungal-bacterial co-occurrence networks in patients with or without MCI

**Fig. 5:**
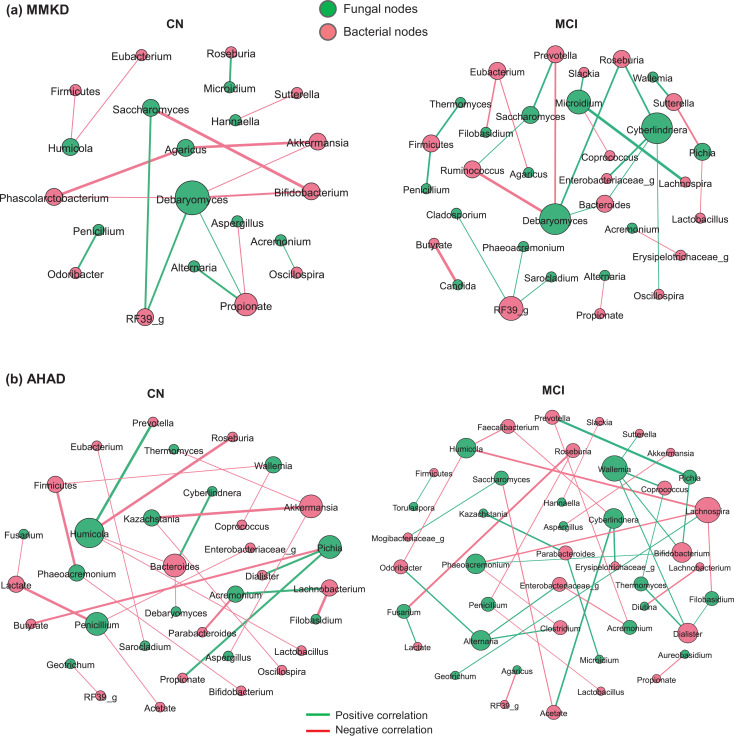
A modified Mediterranean-style ketogenic diet (MMKD) and the American Heart Association Diet (AHAD) differently influence the intestinal inter-kingdom co-occurrence relationships between major fungal and bacterial genera in subjects clinically diagnosed with mild cognitive impairment (MCI) versus cognitively normal (CN) counterparts. Ecological co-occurrence network representing statistically significant correlations (Spearman rho >0.5; p-value <0.05) among differentially abundant fungal and bacterial genera in patients with MCI compared with CN subjects during 6-weeks intervention of (a) MMKD and (b) AHAD intervention.

In addition to the distinct correlation arrays of fungal taxa with CSF markers, we find distinct fungal-bacterial inter-relationship networks in CN subjects versus patients with MCI, wherein these networks differ during MMKD versus AHAD interventions (Fig. 5). Interestingly, compared to CN subjects on MMKD, the MCI subjects on MMKD demonstrate relatively denser array of correlation network (Fig. 5a), suggesting that MMKD modulated the fungal and bacterial microbiome and their co-occurrence more prominently in patients with MCI versus CN subjects. In CN subjects, the network is represented by *Debramyces, Saccharomyces* and *Agaricus* that are linked to bacterial genera including *Akkermansia, Bifidobacterium, Phascolarcobacterium, RF39* and with bacterial metabolite propionate. On the other hand, the co-occurrence network in MCI subjects on MMKD is characterized predominantly by fungal genera *Debramyces, Cyberlindnera, Microidium, Saccharomyces* and *Pichia* that are connected to bacteria taxa *including Ruminococcus, Prevotella, Bacteroides, Roseburia, RF39* and *Lachnospira*. On the other hand, AHAD intervention yields distinct correlation networks between CN and MCI subjects (Fig. 5b) that differ from CN and MCI subjects, respectively, on MMKD (Fig. 5a). The network in CN subjects on AHAD is characterized mainly by *Humicola, Penicillium, Pichia, Acremonum* and *Kazachstania* that correlated mainly to the bacteria genera including *Bacteriodes, Akkermansia* and *Lachnobacterium*. Whereas, the network in MCI subjects is characterized centrally by *Wallemia, Cyberlindnera, Phaeoacremonium, Alternaria* and *Humicola*, which are linked to bacterial genera including *Lachnospira, Parabacteroides, Enterobacteriaceae, Bifidobacterium, Dialister, Roseburia* and *Odoribacter*, thereby indicating a distinct MCI-specific co-occurrence network.

## 4 DISCUSSION

Despite substantial research progress in understanding the AD pathobiology, no effective pharmacologic intervention, other than specific lifestyle and dietary pattern recommendations, is currently available to prevent or ameliorate MCI and AD. In preceding study on this cohort of older adults with metabolic (pre-diabetes) and cognitive (MCI) risk factors for AD [42], we have reported that both MMKD and AHAD diets attained good compliance (>90%) and safety; however, the analysis of magnetic resonance imaging (MRI) and positron emission tomography (PET) scans revealed that only MMKD was able to improve peripheral metabolic profiles, cerebral perfusion, and cerebral ketone body uptake, suggesting the therapeutic potential of such MMKD-style interventions for the prevention of aging-related neurodegenerative disorders. In addition, MMKD led to improved CSF profiles of AD pathological markers as demonstrated by increased Aβ42, decreased total tau, and increased Aβ42/tau ratio, again pointing towards the positive effect of MMKD on CSF AD biomarker profiles [42]. In addition, we reported that these patients with MCI harbor specific gut bacterial signatures that correlate with improved CSF AD biomarker profiles during MMKD intervention [24]. However, the ‘silent microbial population’, the fungal flora (mycobiome), remains largely unexplored in context to host neurological health and diseases. Given that the fungi inhabit symbiotically with bacteria as commensals in our gut, it is perceivable that the mycobiome inadvertently constitute and shape our microbiome. Hence, the investigation of mycobiome in patients with neurodegenerative disorders may provide additional and valuable insight and understanding of yet unidentified mechanisms by which the gut microbiome affect the brain health beyond the bacterial kingdom. In these milieus, considering the emerging notion of the significance of gut microbes beyond bacteria in various aspects of host health and diseases, the present addendum aims to examine the gut mycobiome in these patients with MCI while examining the effects of a MMKD and AHAD intervention on gut mycobiome and its interaction with CSF AD biomarkers and gut bacteriome. To our knowledge, this is the first study demonstrating the fungal microbiome in older adults with MCI versus age-matched CN counterparts and examining the impact of MMKD intervention on gut mycobiome in association with the bacteriome and CSF AD biomarkers.

Although the overall β-diversity does not differ remarkably between the two groups, we find slightly lower Shannon index (a marker of species evenness) in patients with MCI versus CN subjects (Fig. 1a,b). On the other hand, the species richness (total number of species detected) is slightly higher in patients with MCI versus CN subjects (Suppl. Fig. 1a), which is interesting and indicates that patients with MCI may have abnormally higher number or proportion of specific fungal taxa but the mycobiome is still represented predominantly by fewer fungal taxa in these patients versus CN subjects. We have previously reported higher CSF tau levels in patients with MCI versus CN subjects [24] and, interestingly, the total number of fungal taxa are also found to be correlated positively with total tau levels of the CSF of patients with MCI but not of CN subjects (Suppl. Fig. 1c). This is further supported by the LEfSe analysis that reveals a total of nine taxa significantly higher in patients with MCI whereas only two taxa are higher in CN subjects. This indicates a dysbiotic fungal community characterized by over-representation of several taxa in patients with MCI, which, together with MCI-specific gut bacteriome signatures [24], might be associated directly or indirectly with AD pathogenesis. Although several fungal-bacterial taxa appear commonly in co-occurrence networks in CN and MCI subjects (Fig. 1g-h), the modules of these signatures remain distinct between the two groups, indicating differently co-regulated groups of taxa and distinct community partitions between healthy and diseases states. These distinct MCI- and CN-associated networks might hint toward the role of different fungal taxa in MCI phenotype while also indicating the distinct intestinal community partitions that might exist in cognitive impairment, dementia and related milieus. In addition, these analyses suggest that the inter-kingdom ecological co-occurrence relationships may be vital for gut homeostasis in a healthy state whereas gut dysbiosis in a disease state might induce altered bacterial-fungal ecological interactions, which might play a role in AD pathogenesis. In addition to our previous report showing specific gut bacterial signatures in patients with MCI [24], several other recent studies have also reported an altered gut microbiome in patients with AD [21], [22], [23] as well as in several AD animal models [29], [30], [31], [32]. Given that this altered microbiome composition also indicates altered microbiome function, it may be speculated that these specific MCI-specific bacterial and fungal signatures could be playing a contributing role in AD pathogenesis plausibly via altered microbiome metabolism or by modulating intestinal and systemic immunity. In addition, aging as such also involves a reduced gut barrier function, increased gut barrier permeability [65] and dysregulated mucosal surfaces [66], which together with an altered microbiome signatures could also contribute to AD pathology.

In line with our previous report on MMKD-modulated bacterial microbiome [24], the six-week intervention of MMKD induces a more prominent effect on the mycobiome as compared to AHAD (Fig. 2a,c). The MMKD-induced increase in fungal alpha-diversity in patients with MCI but not in their CN counterparts (Fig. 2b) is interesting particularly because the alpha-diversity was lower in patients with MCI at the baseline and was increased (restored) by MMKD in these patients (Fig. 1b). The genus-level mycobiome composition is also modulated more heavily and somewhat positively by MMKD than AHAD intervention (Fig. 2e-g). For instance, the abundance of *Botrytis*, which was higher in patients with MCI at the baseline, is decreased by MMKD in patients with MCI but not in CN subjects, thus hinting toward a positive effect of MMKD on fungal flora, particularly in patients with MCI. Further, the co-occurrence networks lead us to identify distinct co-regulated networks and subnetworks in patients with MCI versus CN subjects, with several fungal and bacterial taxa networking with each other. Although there is an overlap of several central taxa between the two groups, the modularity (way and weight of interaction) of these fungal and bacterial nodes are still quite different in patients with MCI versus CN subjects, indicating a distinct fungal-bacterial interface that might be a potential signature in patients with MCI and AD. Interestingly, compared to CN subjects on MMKD, the patients with MCI on MMKD also demonstrate relatively denser array of correlation network (Fig. 5a), suggesting that MMKD modulated the fungal and bacterial microbiome and their co-occurrence more prominently in patients with MCI versus CN subjects. However, it is worth mentioning here that which of these gut fungi truly colonized the gut of these subjects versus transient fungi that came through diets remains unknown. Many fungal taxa commonly detected in human gut such as *Penicillium, Agaricus, Botrytis* and *Fusarium* are unlikely to remain viable in the gut due to hostile growth conditions or ecological niches and hence are believed to be frequently and transiently introduced through the diet [67, 68]. Nevertheless, the inability to stably colonize the gut may not preclude these fungi from exerting a biological effect on the host or interacting with the gut bacteria. However, this remains one of the limitations of the current study and many other studies using culture-independent methods that the resultant data, despite being high-throughput and wide-ranging in coverage, are unable to distinguish between true colonizers of the gut versus transient members [69, 70]. Several fungi detected in our study including *Candida, Saccharomyces, Penicillium, Cladosporium* and *Aspergillus* have also been frequently detected in several other human studies using culture-dependent methods [70], [71], [72], [73], [74], [75], [76], [77]. However, the role of diet as a direct source of fungi in the human gut remain underexplored. Further studies from a microbial ecology perspective would be indispensable to understand the indigenous versus transient arrays of potential fungal taxa in the human intestinal tract, particularly of those taxa that have sustained influence on host health.

Fungi and bacteria commensally cohabit the human gut and, intrinsically, their mode of interaction in the gut may be altered in a disease state. In this context, our co-occurrence analyses suggest that a complex ecological co-regulation network between fungi and bacteria exists in a healthy gut, which is perturbed in MCI. We notice that the inter-kingdom fungi-bacteria correlation networks are different in patients with MCI versus CN subjects and are affected differently by MMKD versus AHAD (Fig. 5; Suppl. Fig. 2), suggesting that the role of fungi in MCI might be interdependent. In addition, an altered bacterial composition in MCI, as reported in our previous study [24], may lead to different organic environment in the gut, which provides a favorable condition for specific fungal taxa while antagonizing others. A classic example of this is the significant reduction seen in here in the proportion of genus *Candida* in patients with MCI during MMKD intervention (Fig. 3c). The genus *Candida* comprises many opportunistic species implicated in various gut-related diseases including inflammatory bowel diseases, Crohn's disease, ulcerative colitis, and gut inflammation [78]. In addition, there have been a few reports of altered gut fungal community characterized by higher incidences of *Candida* in patients with Autism spectrum disorder [53, 54]. In this context, reduced *Candida* carriage in patients with MCI might reflect another positive outcome of the MMKD intervention. Notably, we find a significantly negative correlation of *Candida* with short-chain fatty acid butyrate (Fig. 5a), a beneficial gut bacterial metabolite which was found to be increased by MMKD in these patients with MCI [24]. One of the ways gut bacteriome keep mycobiome in check is through the production of wide range of small molecules such as SCFAs (e.g., butyrate), which have inhibitory effects against the growth of pathobionts including *Candida albicans* [79, 80]. Together, these data suggest that the MMKD positively modulates the gut microbiome as well as the bacterial metabolites arrays, which in turn may check the overgrowth of opportunistic pathogens such as *Candida*. It should also be an interesting subject for further studies to explore the use of these bacterial metabolites such as butyrate as an alternative to anti-fungal drugs to prevent intestinal colonization of fungal pathobionts, because anti-fungal agents otherwise skews the entire gut mycobiome community while also selecting for drug-resistant strains. A recent study reported that ketogenic diet reduces Th17 cells via reducing gut *Bifidobacterium* population [81]. Interestingly, we also found reduced gut *Bifidobacterium* population following MMKD in these patients with MCI [24]. Butyrate, which was increased by MMKD in these patients with MCI [24], is also known to reduce Th17 cells [82, 83]. Together, these findings suggest that MMKD-induced modulation of host gut microbiome and metabolites may have downstream consequences for host immune or even neuroinflammatory health particularly in neurological disorders including AD. Given the emerging role of neuroinflammation in dementia, cognition and AD [84], [85], [86], these microbial and metabolite modulations may be a potential mechanism contributing to the efficacy of MMKD in improving CSF AD biomarkers in these patients with MCI [24, 42]. Diet-based and microbiome-targeted approaches to prevent or treat AD may have potential translational efficacy. Our previous reports and the present study together show that MMKD can modulate the specific microbiome signatures in association with AD's CSF marker profiles, thereby hinting at the possible therapeutic potential of microbiome in AD. Although whether or not the beneficial effects of MMKD on AD pathological markers are microbiome-mediated and whether there are related associations between the microbiome and Aβ and tau pathology remains unclear.

The present study has several limitations. This was a pilot-scale small sample size observational study and hence the findings may limit generalizable extrapolation and may include confounding biases of certain subgroup factors such as lifestyle, gender, ethnicity and others that might also contribute to distinct mycobiome signatures in the gut. In addition, there might be some carry-over effects of dietary interventions and influences because of the crossover study design. It also remains unclear how these associations between gut fungi, bacteria and AD's CSF profiles relate to AD pathogenesis and whether these associations are cause or consequence of the disease. Further longitudinal studies including pre-symptomatic stages would strengthen our findings and should be able to establish the putative role of microbiome in AD pathogenesis. Covering microbiome alterations at AD stages even earlier than those in the present study cohort might provide further understanding of such causality. In addition, it might also be possible that the alterations in the brain during AD progression could have influenced the microbiome. Although our study finds association of diet-microbiome interaction with AD's CSF markers in patients with MCI, further broader and more inclusive investigations will be required to decipher potential mechanisms and translational implications of these associations with reference to AD pathogenesis and human aging. Such studies would explicate novel microbiome-based prognostic markers of MCI and AD and may lead to discovering novel probiotic microorganisms with therapeutic potential for AD and MCI in future. In summary, our data corroborate the emerging concept of implication of gut-microbiome-brain axis in host neurodegenerative health while fortifying the existing evidence with MCI-specific mycobiome signatures and bacterial-fungal ecologic interactions in context to specific dietary interventions. The findings should facilitate further studies in exploring and developing novel therapeutics aimed at remodeling the gut microbiome functionality to prevent/ ameliorate neurodegenerative disorders including dementia, MCI and AD in high-risk subjects.

## Author contributions

RN: performed bacterial and fungal microbiome measurement, analyzed data, wrote the manuscript; BJN, SC: coordinated in collecting human feces from study participants, revised drafts of manuscript; SW: measured fecal organic acids; SM: helped in correlation network analysis; SC and HY: conceived the idea, supervised the study, helped in data interpretations, revised drafts of manuscript. All authors reviewed and approved the final version of the manuscript.

## Declaration of interests

Dr. Yadav is co-founder and Scientific Research Officer at Postbiotics Inc.; however, no financial and intellectual conflict exist for this work.

## Appendix Supplementary materials

Image, application 1

## References

[1] Global regional (2019). and national burden of Alzheimer's disease and other dementias, 1990-2016: a systematic analysis for the Global Burden of Disease Study 2016. Lancet Neurol.

[2] Hardy J., Allsop D. (1991). Amyloid deposition as the central event in the aetiology of Alzheimer's disease. Trends Pharmacol Sci.

[3] Liu C.C. (2013). Apolipoprotein E and Alzheimer disease: risk, mechanisms and therapy. Nat Rev Neurol.

[4] Penke B., Bogár F., Fülöp L. (2017). β-Amyloid and the Pathomechanisms of Alzheimer's Disease: a Comprehensive View. Molecules.

[5] Le Page A. (2018). Role of the peripheral innate immune system in the development of Alzheimer's disease. Exp Gerontol.

[6] Bronzuoli M.R. (2016). Targeting neuroinflammation in Alzheimer's disease. J Inflamm Res.

[7] Kagan B.L. (2012). Antimicrobial properties of amyloid peptides. Mol Pharm.

[8] Gauthier S. (2006). Mild cognitive impairment. Lancet.

[9] Petersen R.C. (2004). Mild cognitive impairment as a diagnostic entity. J Intern Med.

[10] Panza F. (2005). Current epidemiology of mild cognitive impairment and other predementia syndromes. Am J Geriatr Psychiatry.

[11] Boyle P.A. (2006). Mild cognitive impairment: risk of Alzheimer disease and rate of cognitive decline. Neurology.

[12] Sampson T.R., Mazmanian S.K. (2015). Control of brain development, function, and behavior by the microbiome. Cell Host Microbe.

[13] Wu S.C. (2017). Intestinal microbial dysbiosis aggravates the progression of Alzheimer's disease in Drosophila. Nat Commun.

[14] Kumar D.K. (2016). Amyloid-beta peptide protects against microbial infection in mouse and worm models of Alzheimer's disease. Sci Transl Med.

[15] Sampson T.R. (2016). Gut Microbiota Regulate Motor Deficits and Neuroinflammation in a Model of Parkinson's Disease. Cell.

[16] Berer K. (2017). Gut microbiota from multiple sclerosis patients enables spontaneous autoimmune encephalomyelitis in mice. Proc Natl Acad Sci U S A.

[17] Cekanaviciute E. (2017). Gut bacteria from multiple sclerosis patients modulate human T cells and exacerbate symptoms in mouse models. Proc Natl Acad Sci U S A.

[18] Sharon G. (2019). Human Gut Microbiota from Autism Spectrum Disorder Promote Behavioral Symptoms in Mice. Cell.

[19] Cox L.M., Weiner H.L. (2018). Microbiota Signaling Pathways that Influence Neurologic Disease. Neurotherapeutics.

[20] Ghaisas S., Maher J., Kanthasamy A. (2016). Gut microbiome in health and disease: linking the microbiome-gut-brain axis and environmental factors in the pathogenesis of systemic and neurodegenerative diseases. Pharmacol Ther.

[21] Vogt N.M. (2017). Gut microbiome alterations in Alzheimer's disease. Sci Rep.

[22] Zhuang Z.Q. (2018). Gut Microbiota is Altered in Patients with Alzheimer's Disease. J Alzheimers Dis.

[23] Cattaneo A. (2017). Association of brain amyloidosis with pro-inflammatory gut bacterial taxa and peripheral inflammation markers in cognitively impaired elderly. Neurobiol Aging.

[24] Nagpal R. (2019). Modified Mediterranean-ketogenic diet modulates gut microbiome and short-chain fatty acids in association with Alzheimer's disease markers in subjects with mild cognitive impairment. EBioMedicine.

[25] MahmoudianDehkordi S. (2019). Altered bile acid profile associates with cognitive impairment in Alzheimer's disease-An emerging role for gut microbiome. Alzheimers Dement.

[26] Saji N. (2019). The relationship between the gut microbiome and mild cognitive impairment in patients without dementia: a cross-sectional study conducted in Japan. Sci Rep.

[27] Saji N. (2019). Analysis of the relationship between the gut microbiome and dementia: a cross-sectional study conducted in Japan. Sci Rep.

[28] Alkasir R. (2017). Human gut microbiota: the links with dementia development. Protein Cell.

[29] Shen L., Liu L., Ji H.F. (2017). Alzheimer's Disease Histological and Behavioral Manifestations in Transgenic Mice Correlate with Specific Gut Microbiome State. J Alzheimers Dis.

[30] Bauerl C. (2018). Shifts in gut microbiota composition in an APP/PSS1 transgenic mouse model of Alzheimer's disease during lifespan. Lett Appl Microbiol.

[31] Brandscheid C. (2017). Altered Gut Microbiome Composition and Tryptic Activity of the 5xFAD Alzheimer's Mouse Model. J Alzheimers Dis.

[32] Sanguinetti E. (2018). Microbiome-metabolome signatures in mice genetically prone to develop dementia, fed a normal or fatty diet. Sci Rep.

[33] Solas M. (2017). Inflammation and gut-brain axis link obesity to cognitive dysfunction: plausible pharmacological interventions. Curr Opin Pharmacol.

[34] Zhao Y. (2017). Microbiome-Derived Lipopolysaccharide Enriched in the Perinuclear Region of Alzheimer's Disease Brain. Front Immunol.

[35] Zhao Y., Jaber V., Lukiw W.J. (2017). Secretory Products of the Human GI Tract Microbiome and Their Potential Impact on Alzheimer's Disease (AD): detection of Lipopolysaccharide (LPS) in AD Hippocampus. Front Cell Infect Microbiol.

[36] Welcome M.O. (2018). Current Perspectives and Mechanisms of Relationship between Intestinal Microbiota Dysfunction and Dementia: a Review. Dement Geriatr Cogn Dis Extra.

[37] Claesson M.J. (2012). Gut microbiota composition correlates with diet and health in the elderly. Nature.

[38] Fontana L., Partridge L., Longo V.D. (2010). Extending healthy life span–from yeast to humans. Science.

[39] Schafer M.J. (2015). Calorie Restriction Suppresses Age-Dependent Hippocampal Transcriptional Signatures. PLoS ONE.

[40] Schafer M.J. (2015). Reduction of beta-amyloid and gamma-secretase by calorie restriction in female Tg2576 mice. Neurobiol Aging.

[41] Zhang C. (2013). Structural modulation of gut microbiota in life-long calorie-restricted mice. Nat Commun.

[42] Neth B.J. (2020). Modified ketogenic diet is associated with improved cerebrospinal fluid biomarker profile, cerebral perfusion, and cerebral ketone body uptake in older adults at risk for Alzheimer's disease: a pilot study. Neurobiol Aging.

[43] Sokol H. (2017). Fungal microbiota dysbiosis in IBD. Gut.

[44] Hoarau G. (2016). Bacteriome and Mycobiome Interactions Underscore Microbial Dysbiosis in Familial Crohn's Disease. MBio.

[45] Iliev I.D. (2012). Interactions between commensal fungi and the C-type lectin receptor Dectin-1 influence colitis. Science.

[46] Frykman P.K. (2015). Characterization of Bacterial and Fungal Microbiome in Children with Hirschsprung Disease with and without a History of Enterocolitis: a Multicenter Study. PLoS ONE.

[47] Luan C. (2015). Dysbiosis of fungal microbiota in the intestinal mucosa of patients with colorectal adenomas. Sci Rep.

[48] van der Velden W.J. (2013). Role of the mycobiome in human acute graft-versus-host disease. Biol Blood Marrow Transplant.

[49] Lemoinne S. (2020). Fungi participate in the dysbiosis of gut microbiota in patients with primary sclerosing cholangitis. Gut.

[50] Sharma A. (2019). Associations between fungal and bacterial microbiota of airways and asthma endotypes. J Allergy Clin Immunol.

[51] Chen Y. (2011). Correlation between gastrointestinal fungi and varying degrees of chronic hepatitis B virus infection. Diagn Microbiol Infect Dis.

[52] Zou R. (2020). Dysbiosis of Gut Fungal Microbiota in Children with Autism Spectrum Disorders. J Autism Dev Disord.

[53] Strati F. (2017). New evidences on the altered gut microbiota in autism spectrum disorders. Microbiome.

[54] Iovene M.R. (2017). Intestinal Dysbiosis and Yeast Isolation in Stool of Subjects with Autism Spectrum Disorders. Mycopathologia.

[55] Krikorian R. (2012). Dietary ketosis enhances memory in mild cognitive impairment. Neurobiol Aging.

[56] Thompson L.R. (2017). A communal catalogue reveals Earth's multiscale microbial diversity. Nature.

[57] Caporaso J.G. (2012). Ultra-high-throughput microbial community analysis on the Illumina HiSeq and MiSeq platforms. Isme j.

[58] Schoch C.L. (2012). Nuclear ribosomal internal transcribed spacer (ITS) region as a universal DNA barcode marker for Fungi. Proc Natl Acad Sci U S A.

[59] Nagpal R. (2018). Gut Microbiome Composition in Non-human Primates Consuming a Western or Mediterranean Diet. Front Nutr.

[60] Wang Q. (2007). Naive Bayesian classifier for rapid assignment of rRNA sequences into the new bacterial taxonomy. Appl Environ Microbiol.

[61] Nilsson R.H. (2019). The UNITE database for molecular identification of fungi: handling dark taxa and parallel taxonomic classifications. Nucleic Acids Res.

[62] Craft S. (2017). Effects of Regular and Long-Acting Insulin on Cognition and Alzheimer's Disease Biomarkers: a Pilot Clinical Trial. J Alzheimers Dis.

[63] Claxton A. (2013). Sex and ApoE genotype differences in treatment response to two doses of intranasal insulin in adults with mild cognitive impairment or Alzheimer's disease. J Alzheimers Dis.

[64] Segata N. (2011). Metagenomic biomarker discovery and explanation. Genome Biol.

[65] Nagpal R. (2018). Gut microbiome and aging: physiological and mechanistic insights. Nutr Healthy Aging.

[66] Sato S., Kiyono H., Fujihashi K. (2015). Mucosal Immunosenescence in the Gastrointestinal Tract: a Mini-Review. Gerontology.

[67] Suhr M.J., Hallen-Adams H.E. (2015). The human gut mycobiome: pitfalls and potentials–a mycologist's perspective. Mycologia.

[68] Hallen-Adams H.E., Suhr M.J. (2017). Fungi in the healthy human gastrointestinal tract. Virulence.

[69] David L.A. (2014). Diet rapidly and reproducibly alters the human gut microbiome. Nature.

[70] Nash A.K. (2017). The gut mycobiome of the Human Microbiome Project healthy cohort. Microbiome.

[71] Gouba N., Raoult D., Drancourt M. (2013). Plant and fungal diversity in gut microbiota as revealed by molecular and culture investigations. PLoS ONE.

[72] Gouba N., Raoult D., Drancourt M. (2014). Eukaryote culturomics of the gut reveals new species. PLoS ONE.

[73] Strati F. (2016). Age and Gender Affect the Composition of Fungal Population of the Human Gastrointestinal Tract. Front Microbiol.

[74] Scanlan P.D., Marchesi J.R. (2008). Micro-eukaryotic diversity of the human distal gut microbiota: qualitative assessment using culture-dependent and -independent analysis of faeces. Isme j.

[75] Taylor G.R., Kropp K.D., Molina T.C. (1985). Nine-year microflora study of an isolator-maintained immunodeficient child. Appl Environ Microbiol.

[76] Agirbasli H., Ozcan S.A., Gedikoğlu G. (2005). Fecal fungal flora of pediatric healthy volunteers and immunosuppressed patients. Mycopathologia.

[77] Gouba N., Raoult D., Drancourt M. (2014). Gut microeukaryotes during anorexia nervosa: a case report. BMC Res Notes.

[78] Kumamoto C.A. (2011). Inflammation and gastrointestinal Candida colonization. Curr Opin Microbiol.

[79] Cottier F. (2015). The transcriptional stress response of Candida albicans to weak organic acids. G3 (Bethesda).

[80] Shareck J., Belhumeur P. (2011). Modulation of morphogenesis in Candida albicans by various small molecules. Eukaryot Cell.

[81] Ang Q.Y. (2020). Ketogenic Diets Alter the Gut Microbiome Resulting in Decreased Intestinal Th17 Cells. Cell.

[82] Chen L. (2019). Microbiota Metabolite Butyrate Differentially Regulates Th1 and Th17 Cells' Differentiation and Function in Induction of Colitis. Inflamm Bowel Dis.

[83] Hui W. (2019). Butyrate inhibit collagen-induced arthritis via Treg/IL-10/Th17 axis. Int Immunopharmacol.

[84] Kinney J.W. (2018). Inflammation as a central mechanism in Alzheimer's disease. Alzheimers Dement (N Y).

[85] McGeer E.G., McGeer P.L. (2010). Neuroinflammation in Alzheimer's disease and mild cognitive impairment: a field in its infancy. J Alzheimers Dis.

[86] Heneka M.T. (2015). Neuroinflammation in Alzheimer's disease. Lancet Neurol.

