# Supplementary material for: Gut mycobiome and its interaction with diet, gut bacteria and alzheimer's disease markers in subjects with mild cognitive impairment: A pilot study
Source: eBioMedicine. 2020 Aug 30;59:102950. doi: 10.1016/j.ebiom.2020.102950 (PMC7475073; doi:10.1016/j.ebiom.2020.102950)
Supplement: Supplementary file 1 [file mmc1.pdf]

Supplementary material

Gut mycobiome and its interaction with host diet, gut bacteria and Alzheimer's disease biomarkers in subjects with mild cognitive impairment: a pilot study

Ravinder Nagpal[1,2], Bryan J. Neth[3,4], Shaohua Wang[1,2], Sidharth P. Mishra[1,2], Suzanne Craft[3,*], Hariom Yadav[1,2,*]

[1]*Department of Internal Medicine-Molecular Medicine,* [2]*Department of Microbiology and Immunology, Wake Forest School of Medicine, Winston-Salem, NC, USA.*

[3]*Department of Gerontology and Geriatric Medicine, Wake Forest School of Medicine, Winston-Salem, NC, USA.*

[4]*Department of Neurology, Mayo Clinic, Rochester, MN, USA*

*Correspondence to:

Hariom Yadav, PhD, Assistant Professor, Department of Internal Medicine-Molecular Medicine, Department of Microbiology and Immunology, Center for Diabetes, Obesity and Metabolism, Wake Forest School of Medicine, Wake Forest Biotech Place, Suite 2E-034, 575 Patterson Ave., Winston-Salem, NC 27101, USA. hyadav@wakehealth.edu

Suzanne Craft, PhD, Professor, Gerontology and Geriatric Medicine, J. Paul Sticht Center on Aging, Roena B. Kulynych Center for Memory and Cognition Research, Wake Forest School of Medicine, Winston-Salem, NC 27101, USA. suzcraft@wakehealth.edu

Suppl. Fig. 1

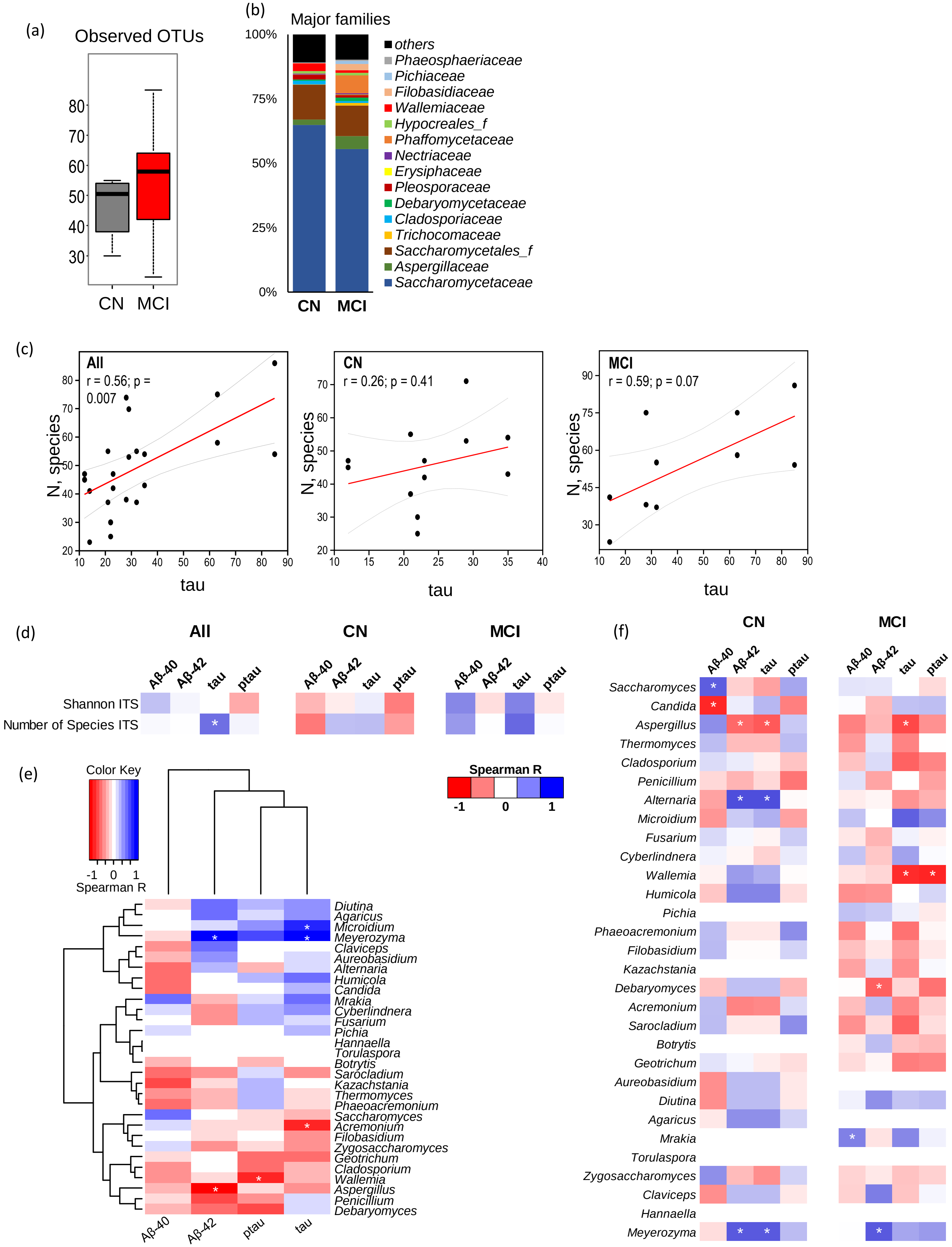

(a)
Observed OTUs
80
70
60
50
40
30
CN
MCI
(b)
Major families
100%
75%
50%
25%
0%
CN
MCI
others
Phaeosphaeriaceae
Pichiaceae
Filobasidiaceae
Wallemiaceae
Hypocreales_f
Phaffomycetaceae
Nectriaceae
Erysiphaceae
Pleosporaceae
Debaryomycetaceae
Cladosporiaceae
Trichocomaceae
Saccharomycetales_f
Aspergillaceae
Saccharomycetaceae
(c)
All
r = 0.56; p = 0.007
N, species
tau
CN
r = 0.26; p = 0.41
MCI
r = 0.59; p = 0.07
(d)
All
CN
MCI
Aβ-40
Aβ-42
tau
ptau
Shannon ITS
Number of Species ITS
Spearman R
-1
0
1
(e)
Color Key
Spearman R
Diutina
Agaricus
Microidium
Meyerozyma
Claviceps
Aureobasidium
Alternaria
Humicola
Candida
Mrakia
Cyberlindnera
Fusarium
Pichia
Hannaella
Torulaspora
Botrytis
Sarocladium
Kazachstania
Thermomyces
Phaeoacremonium
Saccharomyces
Acremonium
Filobasidium
Zygosaccharomyces
Geotrichum
Cladosporium
Wallemia
Aspergillus
Penicillium
Debaryomyces
(f)
CN
MCI
Saccharomyces
Candida
Aspergillus
Thermomyces
Cladosporium
Penicillium
Alternaria
Microidium
Fusarium
Cyberlindnera
Wallemia
Humicola
Pichia
Phaeoacremonium
Filobasidium
Kazachstania
Debaryomyces
Acremonium
Sarocladium
Botrytis
Geotrichum
Aureobasidium
Diutina
Agaricus
Mrakia
Torulaspora
Zygosaccharomyces
Claviceps
Hannaella
Meyerozyma

Suppl. Fig. 2

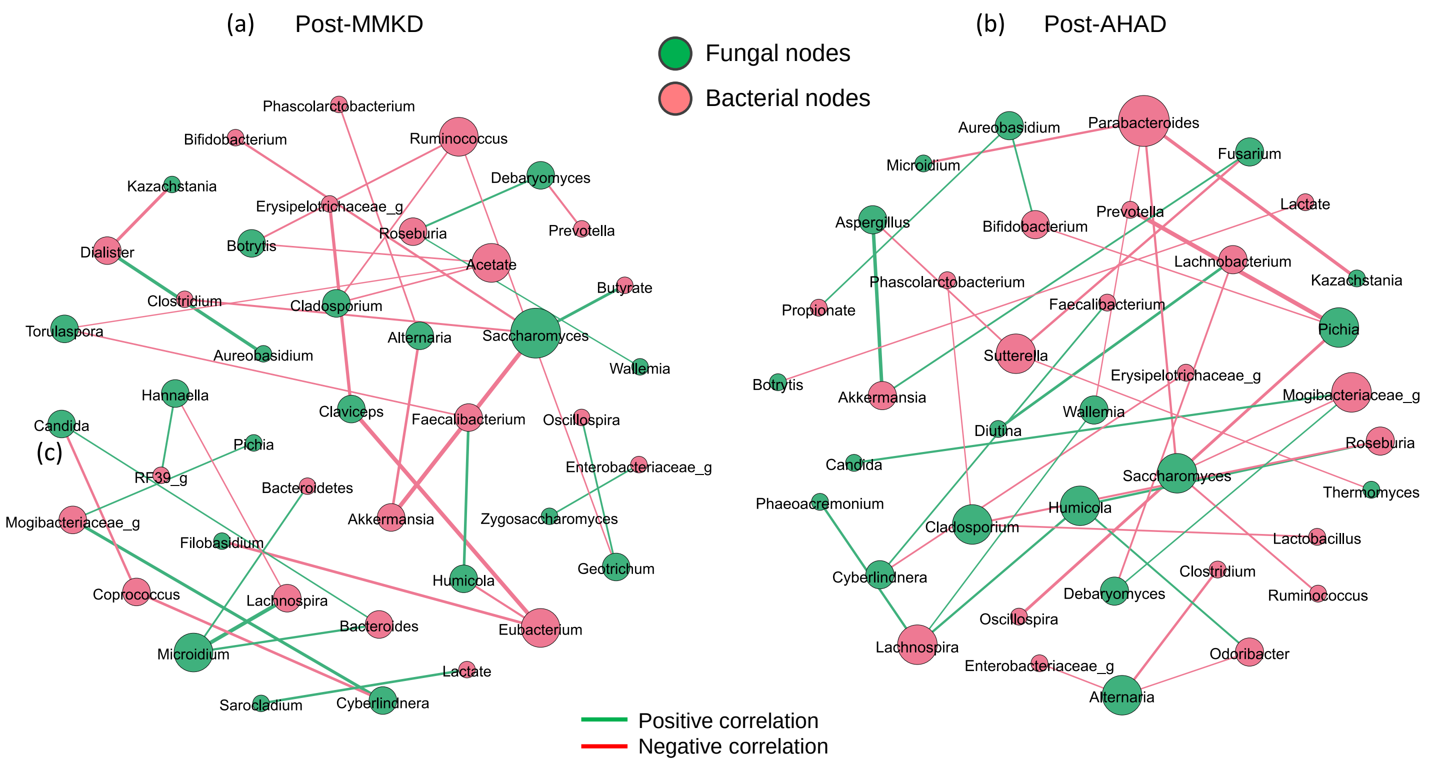

(a) Post-MMKD
(b) Post-AHAD
(c)
Fungal nodes
Bacterial nodes
Positive correlation
Negative correlation
Phascolarctobacterium
Bifidobacterium
Ruminococcus
Kazachstania
Debaryomyces
Erysipelotrichaceae_g
Roseburia
Prevotella
Dialister
Botrytis
Acetate
Butyrate
Clostridium
Cladosporium
Torulaspora
Alternaria
Saccharomyces
Aureobasidium
Wallemia
Hannaella
Claviceps
Faecalibacterium
Oscillospira
Candida
Pichia
RF39_g
Bacteroidetes
Enterobacteriaceae_g
Mogibacteriaceae_g
Akkermansia
Zygosaccharomyces
Filobasidium
Geotrichum
Humicola
Coprococcus
Lachnospira
Bacteroides
Eubacterium
Microidium
Lactate
Sarocladium
Cyberlindnera
Parabacteroides
Fusarium
Lactate
Aspergillus
Lachnobacterium
Phascolarctobacterium
Propionate
Faecalibacterium
Sutterella
Erysipelotrichaceae_g
Botrytis
Diutina
Candida
Phaeoacremonium
Roseburia
Thermomyces
Lactobacillus
Clostridium
Ruminococcus
Oscillospira
Odoribacter
Enterobacteriaceae_g
